# Supplementary material for: Molecular basis of tail-anchored integral membrane protein recognition by the cochaperone Sgt2
Source: J Biol Chem. 2021 Feb 19;296:100441. doi: 10.1016/j.jbc.2021.100441 (PMC8010706; doi:10.1016/j.jbc.2021.100441)
Supplement: Supplemental Figures S1–S5 [file mmc2.pdf]

Fig. S1. Biophysical characterization of the Sgt2-C_{cons} domain. (*A*) CD spectra as in Fig. 1*C* for the conserved C-terminal domains of ySgt2 (blue) and hSgt2 (orange). NMR spectra as in Fig. 1*D* & *E* for ySgt2-C_{cons} (*B*, blue) and hSgt2-C_{cons} (*C*, orange).

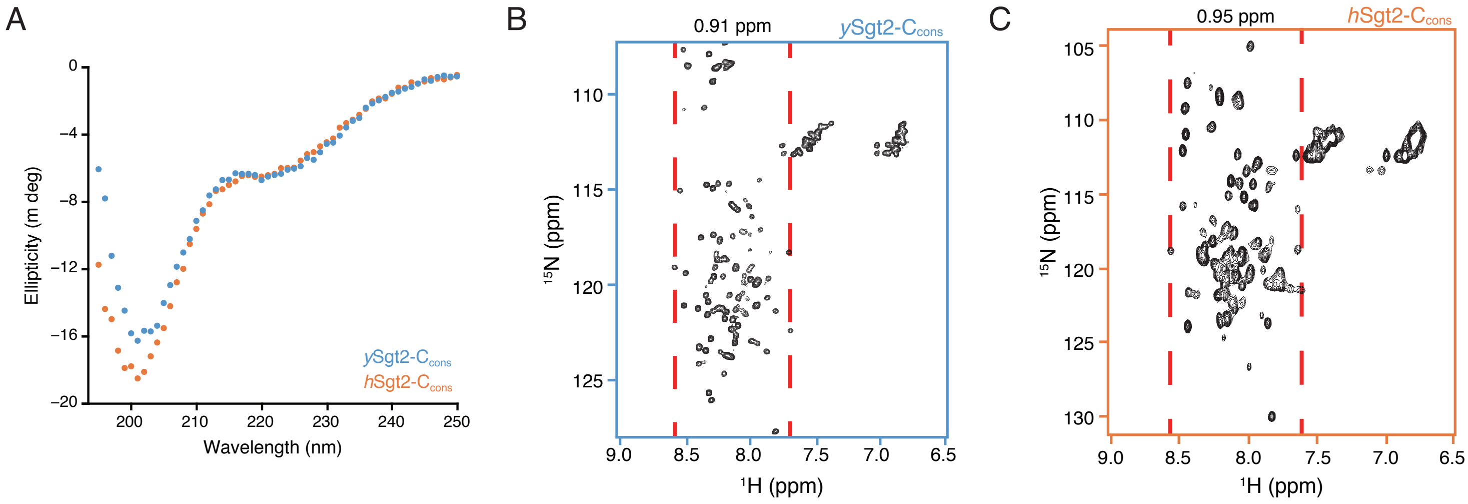
A
0
−4
−8
−12
−16
−20
Ellipticity (m deg)
200
210
220
230
240
250
Wavelength (nm)
ySgt2-Ccons
hSgt2-Ccons
B
0.91 ppm
ySgt2-Ccons
110
115
120
125
15N (ppm)
9.0
8.5
8.0
7.5
7.0
6.5
1H (ppm)
C
0.95 ppm
hSgt2-Ccons
105
110
115
120
125
130
15N (ppm)
9.0
8.5
8.0
7.5
7.0
6.5
1H (ppm)

Figure S1

Fig. S2. Identification of minimal binding region of Sgt2. (*A*) The full image of the gel in Fig 2C. (*B*) An anti-MBP western blot of the lysate from the which the complexes in (A) were purified from. The load concentrations were normalized based on the total optical density of the cells when harvested.

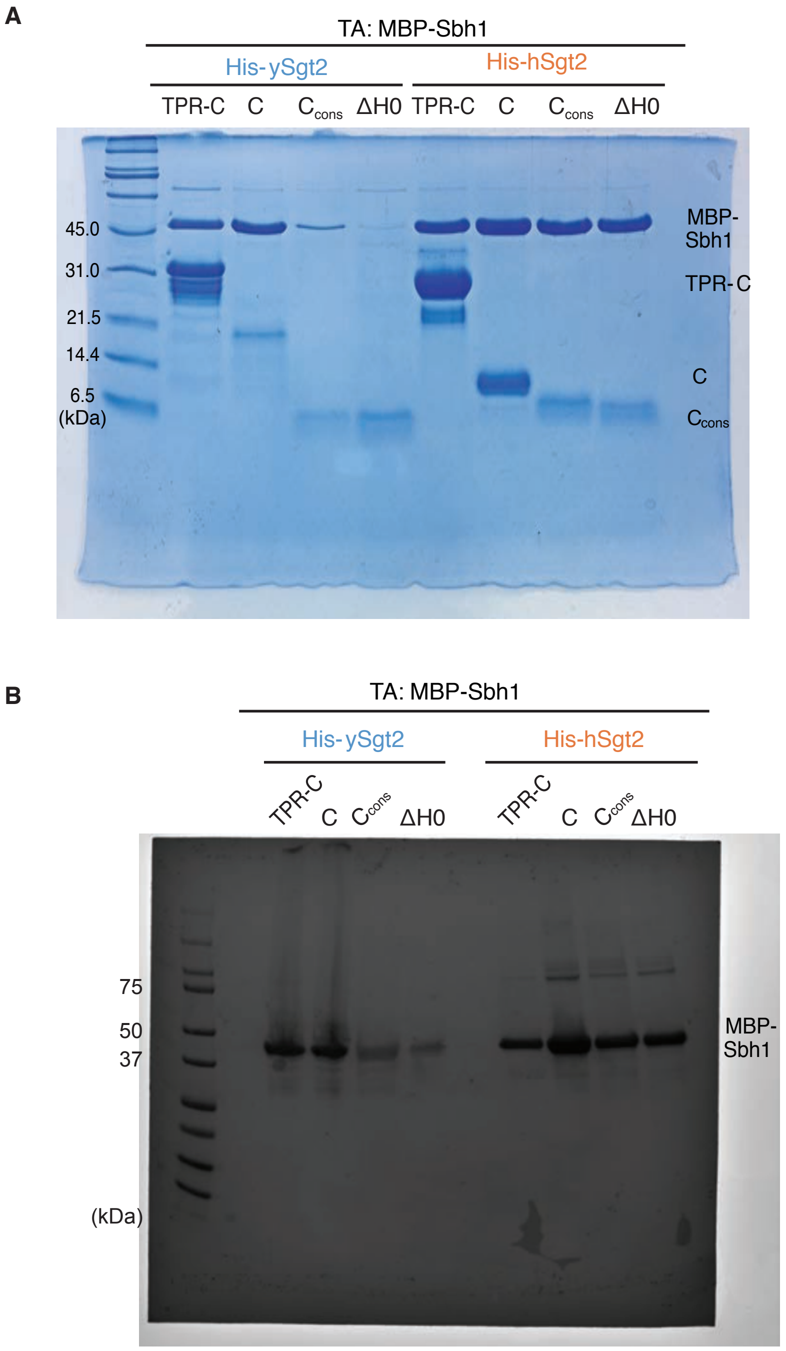
A
TA: MBP-Sbh1
His-ySgt2
His-hSgt2
TPR-C
C
Ccons
ΔH0
TPR-C
C
Ccons
ΔH0
45.0
31.0
21.5
14.4
6.5
(kDa)
MBP-Sbh1
TPR-C
C
Ccons
B
TA: MBP-Sbh1
His-ySgt2
His-hSgt2
TPR-C
C
Ccons
ΔH0
TPR-C
C
Ccons
ΔH0
75
50
37
(kDa)
MBP-Sbh1

Figure S2

Fig. S3. Structural models across prediction methods. (*A*) Predictions from Quark, I-TASSER, Pcons, Phyre2, RaptorX, and Robetta. Methods produce between 5 and 10 models. (*B*) Robetta provides a residue-wise estimated error in Angstroms; this is shown below the corresponding models with a grey bar indicating the C_{cons} region.

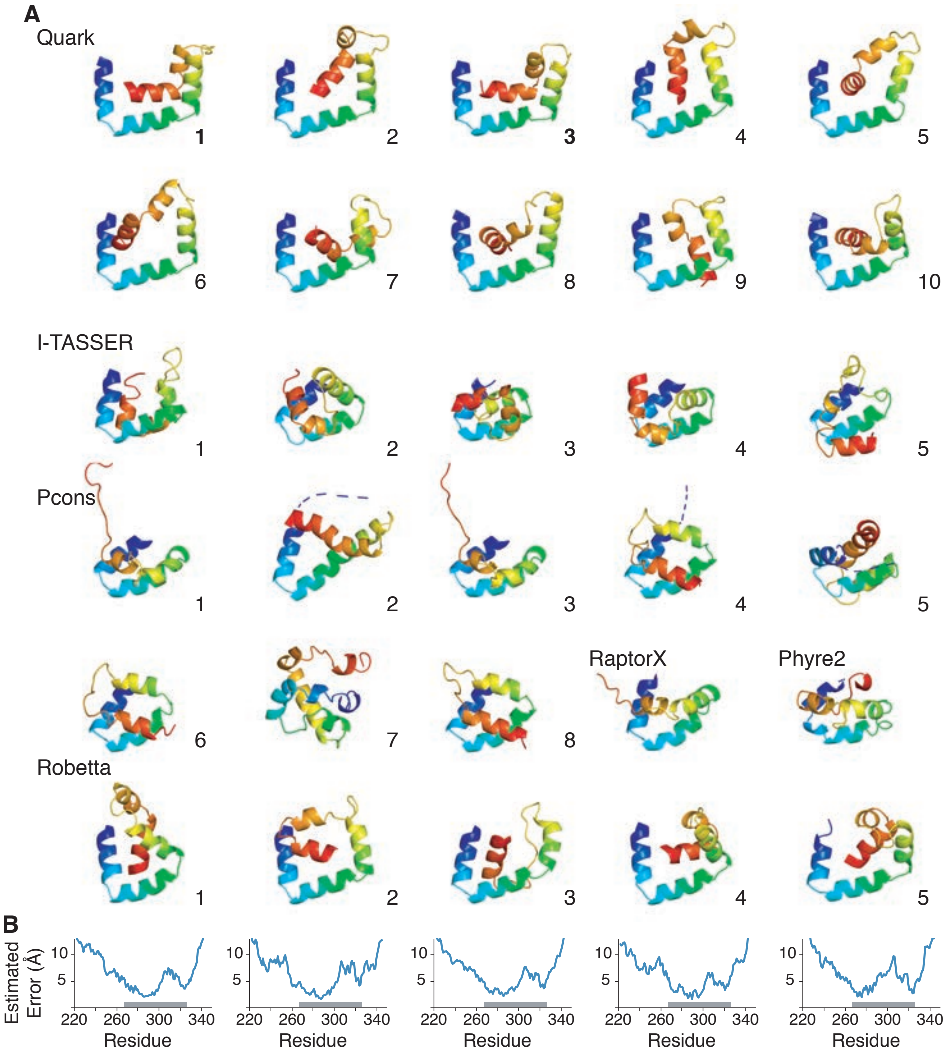

A
Quark
1
2
3
4
5
6
7
8
9
10
I-TASSER
1
2
3
4
5
Pcons
1
2
3
4
5
6
7
8
RaptorX
Phyre2
Robetta
1
2
3
4
5
B
Estimated Error (Å)
10
5
220 260 300 340
Residue

Figure S3

Fig. S4. Cysteine mutants are capable of binding to clients. (*A*) Schematic showing how his-tagged ySgt2-TPR-C and double cysteine mutant constructs were coexpressed with the client 11[L8], and complexes were purified by nickel affinity chromatography. (*B*) A coomassie stained SDS-PAGE gel of the elution fractions demonstrates that 11[L8] was present in the elution suggesting double cysteine mutations do not affect client binding. (*C*) An anti-cMyc western blot of the fractions represented in the SDS-PAGE gel also demonstrates that 11[L8] was present in all eluates.

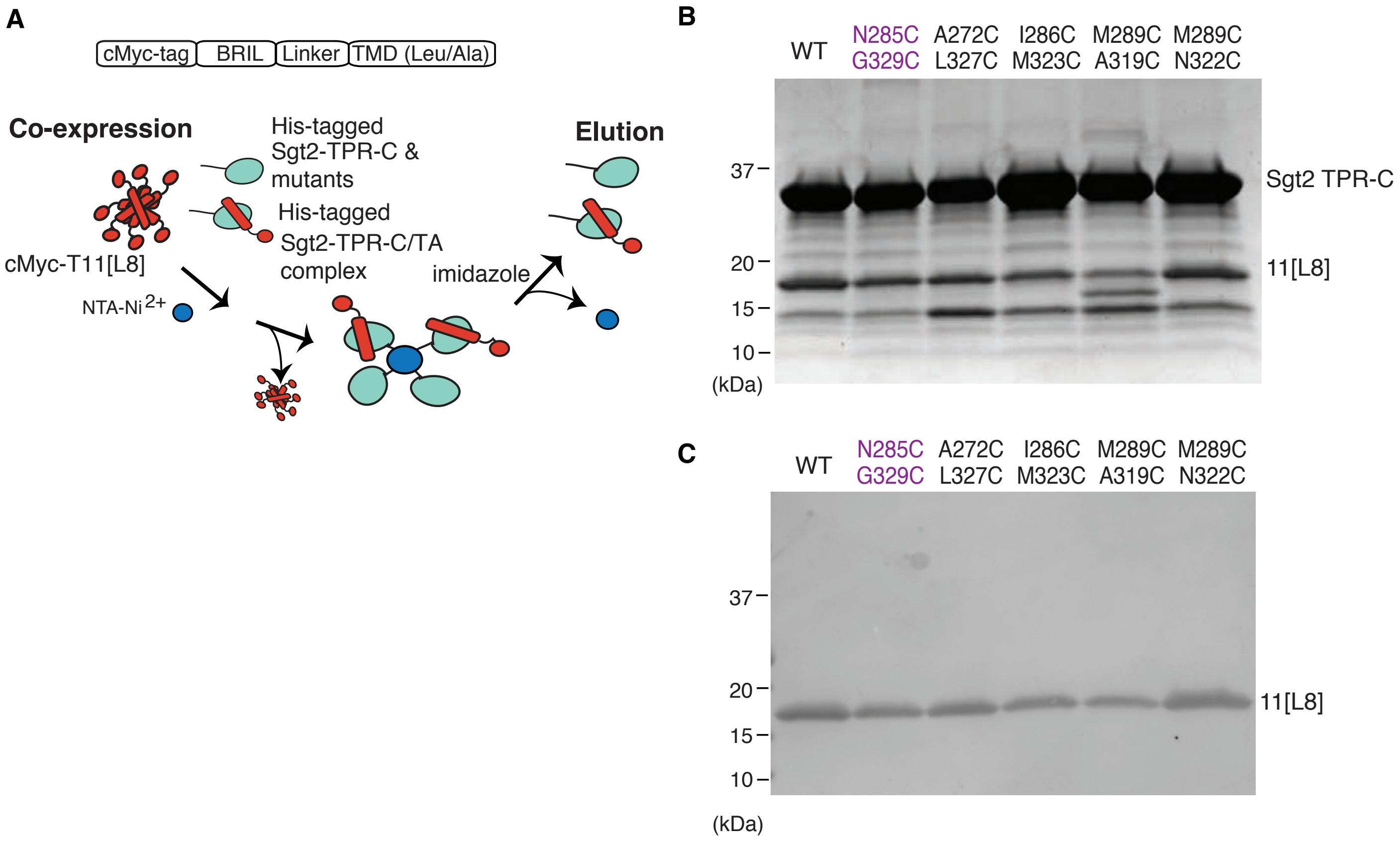
A
cMyc-tag
BRIL
Linker
TMD (Leu/Ala)
Co-expression
His-tagged
Sgt2-TPR-C &
mutants
His-tagged
Sgt2-TPR-C/TA
complex
cMyc-T11[L8]
NTA-Ni 2+
imidazole
Elution
B
WT
N285C G329C
A272C L327C
I286C M323C
M289C A319C
M289C N322C
37
20
15
10
(kDa)
Sgt2 TPR-C
11[L8]
C
WT
N285C G329C
A272C L327C
I286C M323C
M289C A319C
M289C N322C
37
20
15
10
(kDa)
11[L8]

Figure S4

Fig. S5. Distance restraints lead to improved ySgt2-C and suggestive hSgt2-C models. (*A*) Prediction of ySgt2-C using distances from in vitro crosslinking. (*B*) $C_β$-$C_β$ distances between residues probed by *in vitro* disulfide crosslinking for each ySgt2 model. Distances 9 Å or less are colored orange. For models where all distances correspond (4 near and 1 far), the row is shaded grey. (*C*) Models for hSgt2-C using restraints, adding a N-terminal loop, and via the new Robetta TR method.

A ySgt2, using crosslink distances

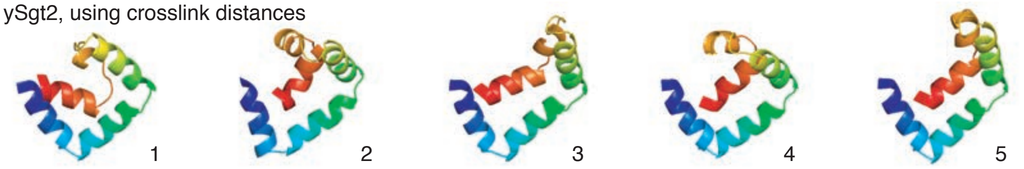

B

	N285-G329	A272-L327	I286-M323	M289-A319	M289-N322
1	18.6	4.6	6.8	7.3	12.4
2	10.3	5.6	5.5	9.0	7.4
3	12.7	6.7	4.8	5.0	8.0
4	9.6	8.2	7.7	9.0	6.7
5	8.5	8.0	7.2	7.2	5.1

C_β-C_β Distance (Å)

9 Å or less

C

hSgt2, using crosslink inferred distances

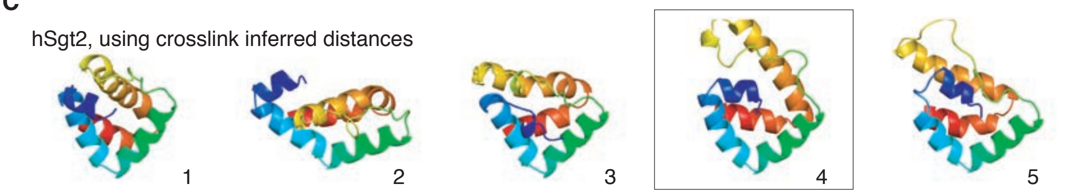

hSgt2, using crosslink inferred distances with N-loop

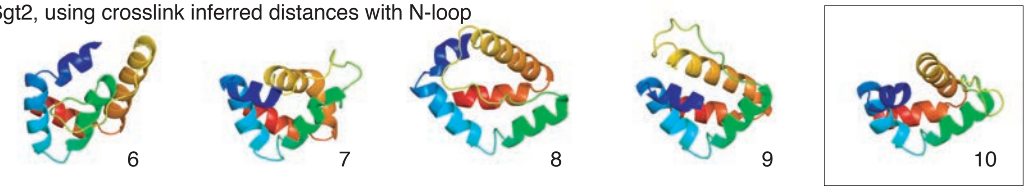

hSgt2, Robetta TR

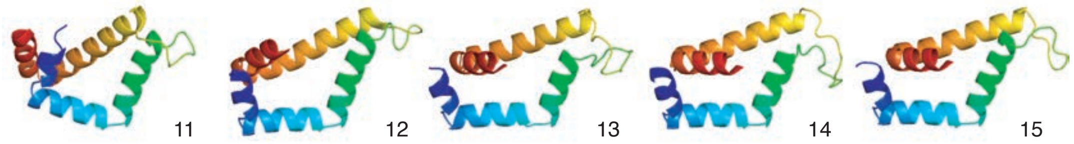

Figure S5
